# Developmental Toxicology of Metal Mixtures in *Drosophila*: Unique Properties of Potency and Interactions of Mercury Isoforms

**DOI:** 10.3390/ijms222212131

**Published:** 2021-11-09

**Authors:** Catherine R. Beamish, Tanzy M. Love, Matthew D. Rand

**Affiliations:** 1 Department of Environmental Medicine, School of Medicine and Dentistry, University of Rochester, Rochester, NY 14642, USA; catherine_beamish@urmc.rochester.edu; 2 Department of Biostatistics and Computational Biology, School of Medicine and Dentistry, University of Rochester, Rochester, NY 14642, USA; tanzy_love@urmc.rochester.edu

**Keywords:** heavy metals, mixture, *Drosophila*, mercury, arsenic, lead, pupariation, metamorphosis, eclosion, toxicokinetics

## Abstract

Mercury ranks third on the U.S. Agency of Toxic Substances and Disease Registry priority list of hazardous substances, behind only arsenic and lead. We have undertaken uncovering the mechanisms underlying the developmental toxicity of methylmercury (MeHg), inorganic mercury (HgCl_2_), lead acetate (Pb), and sodium arsenite (As). To probe these differences, we used the *Drosophila* model, taking advantage of three developmental transitions—pupariation, metamorphosis, and eclosion—to differentiate potentially unique windows of toxicity. We elaborated dose response profiles for each individual metal administered in food and accounted for internal body burden, also extending analyses to evaluate combinatorial metal mixture effects. We observed all four metals producing larval lethality and delayed pupariation, with MeHg being most potent. Compared to other metals, MeHg’s potency is caused by a higher body burden with respect to dose. MeHg uniquely caused dose-dependent failure in eclosion that was unexpectedly rescued by titrating in HgCl_2_. Our results highlight a unique developmental window and toxicokinetic properties where MeHg acts with specificity relative to HgCl_2_, Pb, and As. These findings will serve to refine future studies aimed at revealing tissue morphogenesis events and cell signaling pathways, potentially conserved in higher organisms, that selectively mediate MeHg toxicity and its antagonism by HgCl_2_.

## 1. Introduction

Exposures to toxic metals commonly occur under long-term low-level conditions, and most of these exposures occur as a combination of multiple metals [1]. Variations in exposures have made mixture research a challenging yet pressing topic in the field of toxicology. Metals have been tested in cocktails varying from two to ten constituents [2,3] with higher-order mixtures providing more realistic conditions but making it difficult to distinguish the true interactions between the metals binarily [4,5,6,7]. Additionally, studies investigating metal mixtures have been performed epidemiologically, with little control over the dose and much variation between subjects, making the true nature of the mixture interaction difficult to characterize [8,9]. As a result, investigations into mechanisms causing interactions in metal mixtures need better methodological approaches, including the development of novel experimental models.

At the top of The Agency for Toxic Substances and Disease Registry’s priority list of hazardous substances are the metals arsenic (As), lead (Pb) and mercury (Hg), in that order based on their prevalence in the environment and threat to human health [10]. These metals all share the ability to cause neurotoxicity [11,12,13,14,15] and cause the most harm during development [16]. While a number of studies have addressed the effects of these metals individually and in various combinations, most have been observational and retrospective, lacking the control of a defined experimental model. *Drosophila melanogaster* has long been used as a model to study developmental biology and has been a wellspring for discovery of fundamentally conserved mechanisms of development across higher vertebrates, including humans [17]. The fruit fly has proven particularly effective in studying neurodegenerative diseases [18]. Use of *Drosophila* in toxicology has grown recently, largely because of the ability to implement controlled doses, the ease of scoring multiple developmental landmarks within a short life cycle, and the ability to study a great number of individuals at once [19,20,21,22,23]. Furthermore, the genetic tractability of this model is ideally suited for mechanistic studies. Applications of *Drosophila* to metal toxicology studies have also emerged as a powerful investigative tool [20,22,24,25]. However, only in limited cases has the model been leveraged to investigate metal mixtures in a systemic manner [26].

In the present study, we aimed to perform a comprehensive characterization of the developmental toxicity endpoints across the larval to adult live stages of *Drosophila* for four prominent toxic metal isoforms: methylmercury (MeHg), mercury chloride (HgCl_2_), sodium arsenite (As), and lead (Pb). We implemented a multifactorial design including multiple developmental metrics and endpoints of larval lethality, time to pupariation, pupal lethality, time to metamorphosis, eclosion failure, and time to eclosion. Effects could be easily related to dose at both the level of feeding and internal body burden. We discovered shared and unique attributes of these metals affecting certain endpoints, which highlight the uniquely potent effects of MeHg and the interaction of MeHg with HgCl_2_. 

## 2. Results

### 2.1. Effects of MeHg on Developmental Time Course

**Figure 1:**
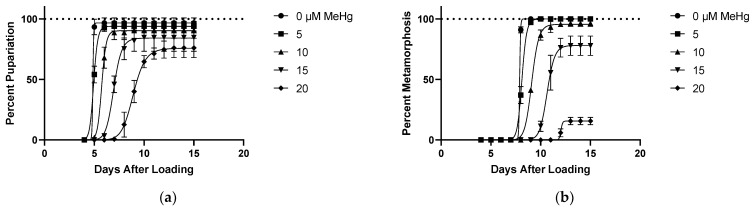

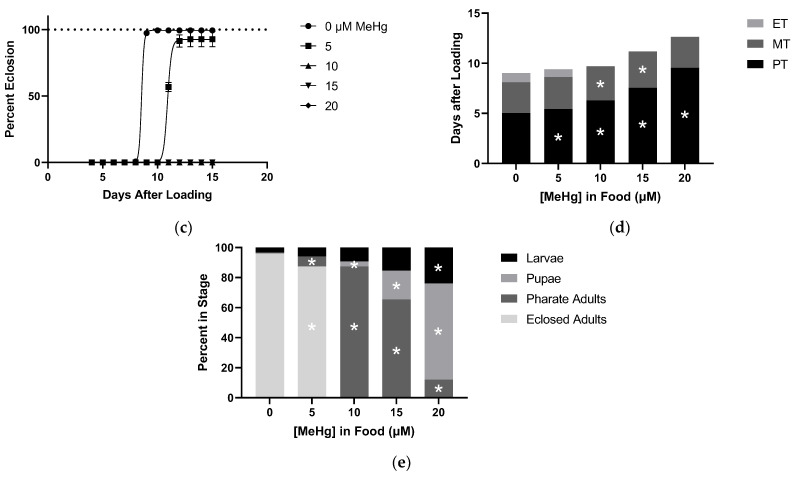
Effects of MeHg on Developmental Time Course. All data from same experiment using CS larvae dosed with indicated concentrations of MeHg in food. (**a**) Pupariation: pupae formation rates of larvae after being loaded onto food. (**b**) Pupa Metamorphosis: pharate adult formation rates of pupae standardized to total pupae formed. (**c**) Pharate Adult Eclosion: rate of eclosion standardized to total number of individuals completing metamorphosis. (**d**) Developmental Timing: representation of time spent in each stage derived from data shown in Figure 1a–c. This allows for accounting of delays seen in later stages caused by carryover of pupariation delays. (**e**) Developmental Endpoints: representation of individuals in each stage at the defined endpoint of 15 days from 1a–c. Individuals that are not eclosed adults correspond to a lethality during development. (* *p* < 0.05 compared to 0 µM, two-way ANOVA).

Larvae reared on various concentrations of MeHg food showed dose-dependent delays in development as shown by the right shift of the time to pupariation curves and quantified by pupariation time (PT, Figure 1a,d). This delay became evident with as low as 10 µM MeHg in the food. Decrease in total animals reaching pupariation, reflecting an increase in larval lethality, was first observed at 20 µM MeHg (Figure 1a,e), where a 20% reduction in animals forming pupae was seen. At this concentration, PT was increased by more than 4 days on average compared to larvae fed on 0 µM food (Figure 1a,d).

When assessing metamorphosis from pupae to the pharate adult stage, we saw delays in the time it took to complete metamorphosis (MT) at doses of 10 µM MeHg and above (Figure 1b,d). Lethality occurring via failure to complete metamorphosis was seen at concentrations as low as 10 µM MeHg dosing, with a dose-dependent increase giving 64% lethality during metamorphosis at 20 µM MeHg (Figure 1b,e).

We next scored eclosion from the pupa casing, which has been noted as the first neuromuscular behavior the adult fly performs. A complete failure in eclosion was seen with doses of 10 µM MeHg or higher in the food (Figure 1c). Given this lethality at 10 µM MeHg, we could only calculate an eclosion time (ET) for 0 µM and 5 µM MeHg treatments, which showed no delay despite the carryover of an approximate 2 day PT delay (Figure 1c,d). Additionally, with 5 µM MeHg treatment, a significant amount of eclosion failure occurred (6%), whereas the vast majority of individuals at 10 µM MeHg were halted in the pharate adult stage (Figure 1c,e). These data indicated eclosion behavior to be the most sensitive transition for MeHg-induced developmental lethality, with the next most sensitive stage being metamorphosis. Pupariation was seen to be the stage least affected by MeHg toxicity.

### 2.2. Effects of HgCl_2_ on Developmental Time Course

**Figure 2:**
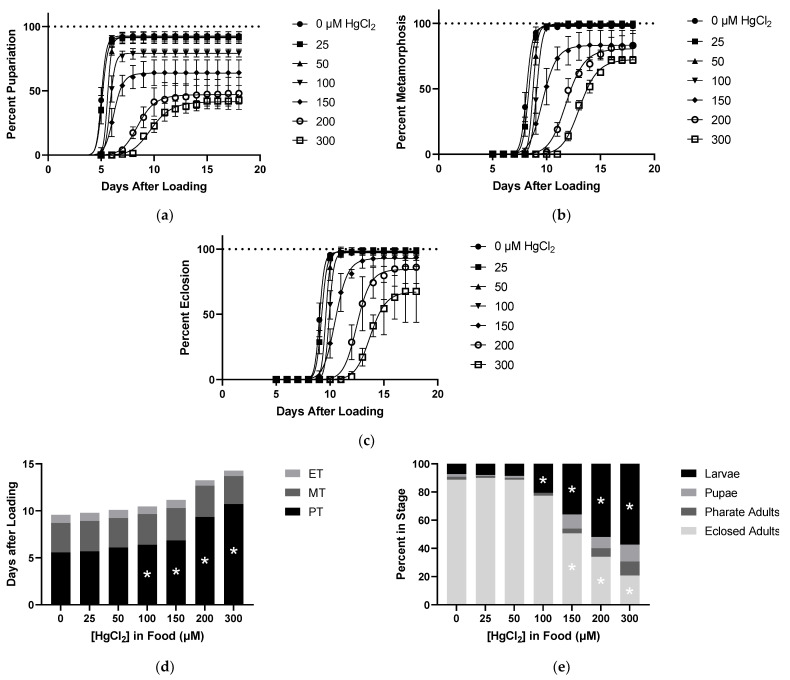
Effects of HgCl_2_ on Developmental Time Course. All data from same experiment using CS larvae dosed with indicated concentrations of HgCl_2_ in food. (**a**) Pupariation: pupae formation rates of larvae after being loaded onto food. (**b**) Pupa Metamorphosis: pharate adult formation rates of pupae standardized to total pupae formed. (**c**) Pharate Adult Eclosion: rate of eclosion standardized to total number of individuals completing metamorphosis. (**d**) Developmental Timing: representation of time spent in each stage derived from data shown in Figure 1a–c. This allows for accounting of delays seen in later stages caused by carryover of pupariation delays. (**e**) Developmental Endpoints: representation of individuals in each stage at the defined endpoint of 18 days from 1a–c. Individuals that are not eclosed adults correspond to a lethality during development. (* *p* < 0.05 compared to 0 µM, two-way ANOVA).

Larvae reared on various concentrations of HgCl_2_ food showed dose-dependent delays in development, as shown by the right shift of the time to pupariation curves and increased PT (Figure 2a,d). Larval lethality was also apparent with food doses of 100 µM HgCl_2_ and above (Figure 2a,e). For HgCl_2_, there were no delays seen in MT despite carryover of PT delay, and likewise, no delays in ET were seen (Figure 2b–d). In addition, there was no significant lethality during the pupal stage nor failure of pharate adult eclosion when accounting for the carryover lethality from the larval stage (Figure 2b,c,e). The main developmental phenotypes of HgCl_2_ included pupariation delay and larval lethality, initially appearing at the same dose (100 µM), thus highlighting the sensitivity of earlier developmental stages to HgCl_2_ toxicity.

### 2.3. Effects of As on Developmental Time Course

**Figure 3:**
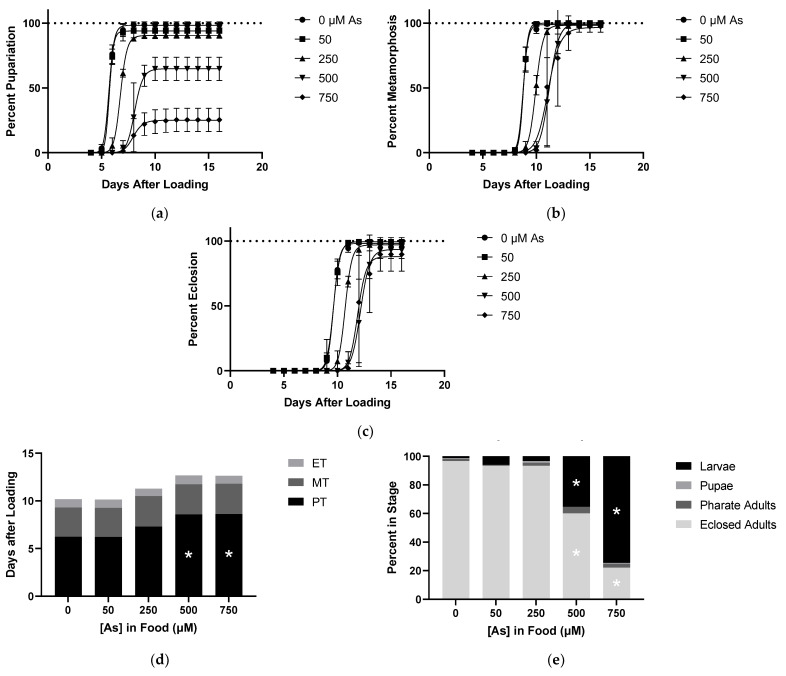
Effects of As on Developmental Time Course. All data from same experiment using CS larvae dosed with indicated concentrations of As in food. (**a**) Pupariation: pupae formation rates of larvae after being loaded onto food. (**b**) Pupa Metamorphosis: pharate adult formation rates of pupae standardized to total pupae formed. (**c**) Pharate Adult Eclosion: rate of eclosion standardized to total number of individuals completing metamorphosis. (**d**) Developmental Timing: representation of time spent in each stage derived from data shown in Figure 1a–c. This allows for accounting of delays seen in later stages caused by carryover of pupariation delays. (**e**) Developmental Endpoints: representation of individuals in each stage at the defined endpoint of 16 days from 1a–c. Individuals that are not eclosed adults correspond to a lethality during development. (* *p* < 0.05 compared to 0 µM, two-way ANOVA).

Larvae reared on various concentrations of As food showed dose-dependent delays in development, as shown by the right shift of the time to pupariation curves and increased PT (Figure 3a,d) Larval lethality was also apparent with food doses of 500 µM As and above. There were no delays in MT despite carryover of PT delay and similarly no delays in ET (Figure 3b–d). For As, there was no lethality during the pupal stage nor failure of pharate adult eclosion when accounting for the carryover lethality at the larval stage (Figure 3b,c,e). This experiment identified the primary effects of As to be pupariation delay and larval lethality, similar to HgCl_2_.

### 2.4. Effects of Pb on Developmental Time Course

**Figure 4:**
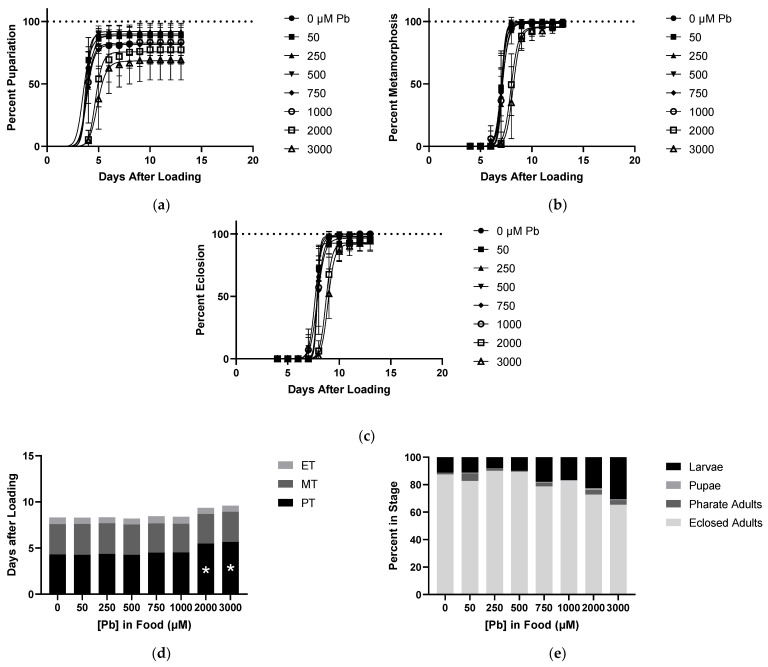
Effects of Pb on Developmental Time Course. All data from same experiment using CS larvae dosed with indicated concentrations of Pb in food. (**a**) Pupariation: pupae formation rates of larvae after being loaded onto food. (**b**) Pupa Metamorphosis: pharate adult formation rates of pupae standardized to total pupae formed. (**c**) Pharate Adult Eclosion: rate of eclosion standardized to total number of individuals completing metamorphosis. (**d**) Developmental Timing: representation of time spent in each stage derived from data shown in 1a–c. This allows for accounting of delays seen in later stages caused by carryover of pupariation delays. (**e**) Developmental Endpoints: representation of individuals in each stage at the defined endpoint of 13 days from 1a–c. Individuals that are not eclosed adults correspond to a lethality during development. (* *p* < 0.05 compared to 0 µM, two-way ANOVA).

Larvae reared on various concentrations of Pb began to show delays in time to pupariation, but food concentrations of 2000 µM were needed before this effect was observed (Figure 4a,d). At this high concentration producing small delays in PT, a trend toward larval lethality was detected but lacked significance (Figure 4a,e). There were also no toxic effects from Pb impacting MT, ET, or lethality during metamorphosis and eclosion (Figure 4b–e).

### 2.5. Dose Response with Respect to Body Burden for MeHg, HgCl_2_, As, and Pb

**Figure 5:**
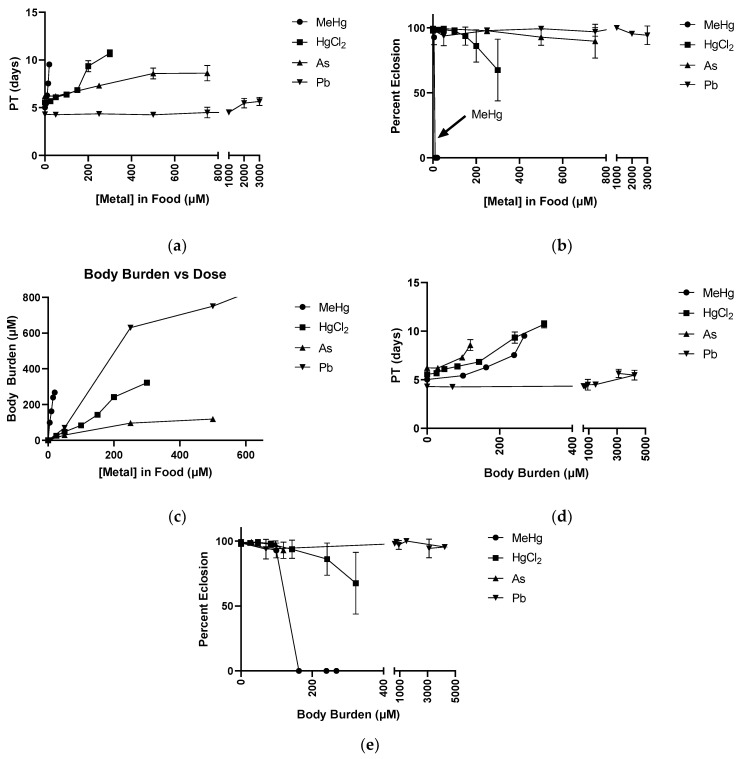
Dose response with respect to body burden for MeHg, HgCl_2_, As, and Pb. (**a**) Pupariation Time (PT) versus Food Dose: pupariation delays induced by all four metals at concentration ranges tested. (**b**) Pharate Adult Eclosion versus Food Dose: eclosion failure induced by all four metals at concentration ranges tested. (**c**) Body Burden versus Food Dose: internal dose of each metal for each treatment in food. A more efficient accumulation of each metal is represented by a steeper slope. (**d**) PT versus Body Burden: pupariation delays caused by each metal while accounting for internal dose. (**e**) Pharate Adult Eclosion versus Body Burden: internal dose of each metal impacts the ability to complete eclosion.

The dose response of delays in PT and of failure in eclosion demonstrated a toxicity hierarchy with MeHg being most potent, followed by HgCl_2_, As, then Pb (Figure 5a,b). We next wanted to investigate the relationship of the internal dose, or body burden, of each metal with the effect on development. Body burden of each metal was determined on a weight basis (ppm) and expressed in molar concentration assuming 1 g of body weight to be equal to 1 mL of volume.

The plot of body burden versus the dose in the food shows that MeHg had the steepest slope, demonstrating the ability of flies to accumulate MeHg at a level averaging 16-fold over what was present in the food (Figure 5c). Pb body burden levels also demonstrated accumulation, but to a lesser extent, of a 1.6-fold increase in internal Pb compared to that in the food. In contrast, HgCl_2_ produced a body burden in the fly essentially equivalent in concentration to that in the food (Figure 5c). Lastly, As was not accumulated efficiently, such that the body burden only averaged 0.4 times that of the food’s concentration of the metal (Figure 5c).

We next examined how PT changes with respect to body burden. Interestingly, the prior hierarchy of MeHg, HgCl_2_, and As was reversed, whereby As showed a slightly more potent effect on PT with increasing body burden as compared to HgCl_2_ and MeHg (Figure 5d), yet the differences in their linear regression slopes did not reach significance. However, with inhibition of eclosion, MeHg continued to demonstrate a much more potent effect relative to HgCl_2_, As, and Pb when evaluated with body burden (Figure 5e).

### 2.6. Effects of MeHg and HgCl_2_ Mixtures on Developmental Time Course

Given the unique and potent toxicity profile of MeHg on eclosion, we chose to systematically evaluate effects of binary mixtures containing MeHg, beginning with HgCl_2_. Within the combinations, several dose levels of each metal were evaluated, spanning concentrations where no effects were seen to levels where we previously identified larval lethality and pupariation delay, as well as metamorphosis and eclosion failure in the case of MeHg.

**Figure 6:**
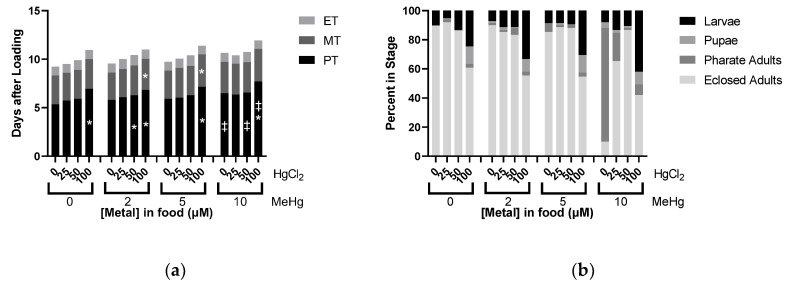
Effects of MeHg and HgCl_2_ mixtures on Developmental Time Course. All data from same experiment using CS larvae dosed with indicated concentrations of MeHg and HgCl_2_ in food. (**a**) Developmental Timing: average time spent in each stage across different treatment conditions. (**b**) Developmental Endpoints: average of individuals remaining in each stage of development at endpoint of 16 days. (* *p* < 0.05 compared to fixed value of MeHg at 0 µM HgCl_2_, ^‡^ *p* < 0.05 compared to fixed value of HgCl_2_ at 0 µM MeHg, two-way ANOVA).

We observed an additive dose response in PT and MT when combining the two metals in the food, where adding MeHg produced delays at 50 µM and 100 µM HgCl_2_ not seen without MeHg, respectively (Figure 6a). No changes in ET were measurable across either metal’s concentration range (Figure 6a). Remarkably, analysis of developmental endpoints demonstrated a strong HgCl_2_ antagonism of MeHg’s toxicity phenotype of eclosion failure (Figure 6b). This was evident when looking at the 10 µM MeHg fixed dose with titration of HgCl_2_ between 0–100 µM (Figure 6b). Where 10 µM MeHg alone caused near complete failure of eclosion (most animals stall at pharate adult stage), addition of HgCl_2_ to the 50 µM level resulted in a nearly complete rescue of eclosion. In contrast, there was no evidence that HgCl_2_’s larval lethality phenotype was enhanced or inhibited in combinations with MeHg (Figure 6b).

### 2.7. Effects of MeHg and As Mixtures on Developmental Time Course

**Figure 7:**
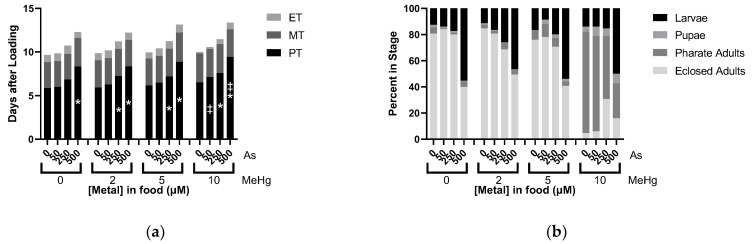
Effects of MeHg and As mixtures on Developmental Time Course. All data from same experiment using CS larvae dosed with indicated concentrations of MeHg and As in food. (**a**) Developmental Timing: average time spent in each stage across different treatment conditions. (**b**) Developmental Endpoints: average of individuals remaining in each stage of development at endpoint of 16 days. (* *p* < 0.05 compared to fixed value of MeHg at 0 µM As, ^‡^ *p* < 0.05 compared to fixed value of As at 0 µM MeHg, two-way ANOVA).

Interactions of As and MeHg were then evaluated similarly as above with HgCl_2_. Similar results were seen where PT showed an additive dose response as MeHg caused more potent delays at 250 µM As levels in the food, and As combined with 10 µM MeHg also induced delays not present without As (Figure 7a). No interactions of MeHg and As were seen with MT and ET (Figure 7a). A subtle antagonistic impact of As on MeHg’s eclosion failure phenotype was also observed, which was only seen in the 10 µM MeHg fixed dose group, yet MeHg did not impact the larval lethality caused by As.

### 2.8. Effects of MeHg and Pb mixtures on Developmental Time Course

**Figure 8:**
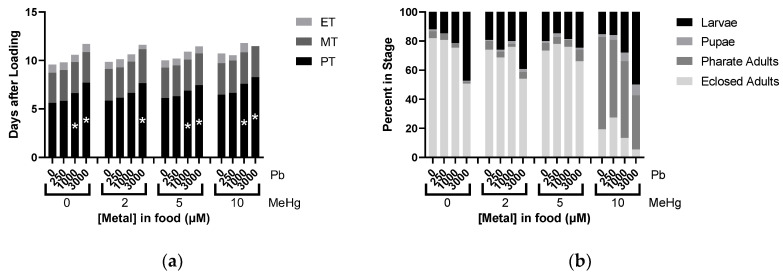
Effects of MeHg and Pb mixtures on Developmental Time Course. All data from same experiment using CS larvae dosed with indicated concentrations of MeHg and Pb in food. (**a**) Developmental Timing: average time spent in each stage across different treatment conditions. (**b**) Developmental Endpoints: average of individuals remaining in each stage of development at endpoint of 16 days. (* *p* < 0.05 compared to fixed value of MeHg at 0 µM Pb, two-way ANOVA).

Interactions of Pb and MeHg were then evaluated similarly as above with HgCl_2_. No interactions were seen with PT in the Pb and MeHg mixture conditions, and we again saw no effect on MT and ET (Figure 8a). No significant antagonism or synergy was found between the metals in the developmental endpoints we observed.

### 2.9. Eclosion and Body Burden Assessment of MeHg and HgCl_2_ Mixtures

**Figure 9:**
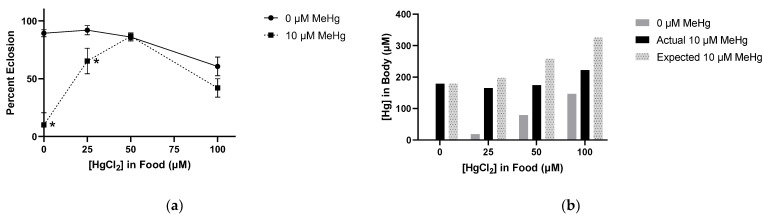
Eclosion and Body Burden Assessment of MeHg and HgCl_2_ Mixtures. (**a**) Eclosion: experimental data from Figure 6 represented as ability to complete eclosion at varying levels of HgCl_2_ while fixing MeHg food concentration at 0 µM or 10 µM. (**b**) Body Burden Assessment: Internal dose of total Hg of pupae after exposure to MeHg and HgCl_2_ mixtures in food alongside what might be expected from adding the body burdens of individuals treated with each metal individually. (* *p* < 0.05 compared to fixed HgCl_2_ dose at 0 µM MeHg, two-way ANOVA).

Our above results indicated a curious antagonistic effect of HgCl_2_ on MeHg toxicity. We examined this further by relating the overall effects on eclosion with body burden of Hg. Plotting the above data in a different format, we saw that HgCl_2_ added to larvae reared on 10 µM MeHg food showed a biphasic response (Figure 9a). Addition of HgCl_2_ up to 50 µM showed a rescue in eclosion behavior, whereas 100 µM HgCl_2_ reverted the rescue to decrease the survival rate. Interestingly, the decrease in eclosion rates at these latter combined MeHg/HgCl_2_ concentrations was statistically equivalent to rates seen with HgCl_2_ alone, indicating the effects of MeHg had been alleviated (Figure 9a). In parallel, we examined the total Hg body burden. We anticipated that the body burden should reflect the sum of accumulated MeHg and HgCl_2_ at each combined dose amount if there were no toxicokinetic interactions. We saw that with combined exposures, the total Hg values were far less than expected from the values derived from each metal applied individually. This profile indicated a true antagonism by ANCOVA and by Bliss and Loewe analyses (not shown), whereby one of the metal species was causing a diminished absorption or enhanced excretion of the other.

## 3. Discussion

We leveraged the *Drosophila* model to allow for the investigation of toxicants’ impact on development by themselves and as mixtures. First, we were able to determine relevant doses and phenotypes for each of the four metal species. Through a series of developmental time course experiments, we found MeHg to display unique toxicity phenotypes, causing eclosion failure with failure and delays in metamorphosis also present. These findings are consistent with MeHg having unique toxicodynamic interactions occurring during development and contributing to the flies’ inability to eclose. At the same time, all of the metals were shown to delay pupariation, but MeHg showed extreme potency 10-fold higher than the next most potent metal. We demonstrated that body burden accounts for MeHg’s potency in stalling pupariation, such that MeHg, HgCl_2_, and As all showed no difference in potency when accounting for body burden. This may point to a common mechanistic target or pathway with respect to larval growth and pupariation shared between the metals we studied. However, MeHg showed unique potency in preventing pharate adult eclosion. This exclusive toxicity profile of MeHg was retained after analyzing the body burden and points to the existence of a uniquely sensitive toxicity target for MeHg. With numerous developmental pathways being highly conserved between flies and mammals, these data provide insight and an approach to defining specific target(s) of MeHg relevant to humans.

A remarkable finding was the apparent antagonism of HgCl_2_ toward MeHg toxicity, reflected by a rescue of MeHg inhibition of eclosion behavior. Accounting for body burden suggested that the metals are antagonistic at the level of uptake. One possibility to explain this antagonism is the ability of Hg^2+^ ions to block the L-type large neutral amino acid transporters (LATs). MeHg has been shown to be transported across cells via LATs using a molecular mimicry of methionine when MeHg is conjugated to the free amino acid cysteine (Cys) [27,28]. Typical substrates of LATs include methionine, histidine, phenylalanine, leucine, isoleucine, valine, tyrosine, and tryptophan [29]. HgCl_2_ has been found to noncompetitively inhibit LAT transport of histidine and phenylalanine by preventing necessary conformational changes [30,31]. We infer that HgCl_2_ acts similarly here to noncompetitively inhibit uptake of MeHg-Cys conjugates via LATs, leading to a lower body burden of MeHg seen when treating with a mixture of the two metals. The implications of this antagonism are potentially influential for human MeHg exposures. It has been shown that MeHg can be demethylated to Hg^2+^ in the gut lumen, presumably by resident bacteria [32,33]. This biotransformation may thus yield an enhanced resistance to MeHg uptake, as the Hg^2+^ product could serve to not only be more poorly absorbed, but also act as an antagonist to MeHg absorption. 

Relative to MeHg, much lower potency was seen with HgCl_2_ and As. However, it was apparent that the toxicity of these metals was more closely related when considering effects relative to the internal dose (body burden) and the specific developmental stage that was affected, i.e., pupariation. Pupariation is dependent on two fundamental processes: larval growth to a sufficient size and implementation of hormonal signaling to induce the transition to pupa formation. Both of these processes are elaborately controlled by a number of highly conserved developmental pathways, namely the insulin-like peptide (Dilp8-Lgr3) pathway for overall growth [34] and the ecdysone hormonal pathway for pupariation [35]. While these three metals yield similar gross phenotypes of PT, the model established here offers the opportunity to parse out more metal-specific targets in the future by leveraging the molecular genetic tools of the fly model [24].

Perhaps a limitation of the study and the fly model was the surprising finding that Pb was ineffective in impairing development of *Drosophila*; this could not be attributed to an inability of the flies to absorb the metal. Pb was still able to be concentrated in the flies at 1.6 times the dose given in the food but did not produce the expected developmental delays or lethality based on trends seen with the other three metals. One possibility for this could be sequestration of Pb into a compartment away from the target site that induces pupariation delays. This may be consistent with how Pb acts toxicokinetically in humans, where it is quickly sequestered to bone [36]. Thus, future studies of Pb with the fly model should take into consideration the possibility for a unique profile of distribution in this model, which could influence the interpretation of Pb’s toxicity. 

Another limitation of this study is that, with respect to dose, it is difficult to translate effects seen in the *Drosophila* model to relevant doses in rodent or humans. It has been common laboratory practice to use higher-than-relevant doses of toxicants for mechanistic study [37]. Nonetheless, a point of reference for MeHg is that 5µM is equivalent to 1 ppm in the food source. In the U.S., mercury levels in fish are estimated to range from 0.3–3 ppm [38]. However, in contrast to humans, the larva feed continually on the MeHg food and reach a much-elevated steady state, such that 5 µM feeding gives ~20 ppm (100 µM) body burden [22]. In human cases where MeHg poisoning had occurred, neurological effects were observable at levels equivalent to ~10 ppm in blood (~50 µM), only a 2-fold difference from that given by our lowest dose that showed delays in pupariation [39,40]. Our studies herein have shown continuous feeding of larvae on 50 µM As gives a total body burden of ~30 µM, corresponding to ~2 ppm. Comparably, studies of water poisonings in Bangladesh and Taiwan have attributed As levels reaching 0.71 and 1.82 ppm, respectively [41,42,43]. In Bangladesh, levels in human hair reached up to 14.91 ppm, which is higher than the levels of ~10 ppm (120 µM) reached in our experiment feeding 500 uM As food and producing measurable deficits in development [42]. Additionally, since the distribution of the metals in the developing flies remains to be characterized, it is difficult to relate body burden to the level of metal at the site of toxic action (e.g., brain). This assessment will require further study. 

## 4. Materials and Methods

### 4.1. Drosophila Stocks

The *Drosophila* strain, Canton S (CS, #1), was obtained from the Bloomington Drosophila Stock Center (Indiana University, Bloomington, IN, USA). Flies were kept on a 12/12 h light/dark cycle in a 25 °C humidified chamber on a standard fly food made of cornmeal, molasses, yeast, and agar. 

### 4.2. Developmental Time Course and Body Burden Measurements

Canton S embryos were collected within a 12 h laying period from a mating population of 150–300 flies, then aged to larvae at 25 °C. First instar Canton S larvae were transferred to vials of food (Jazz Mix, Fisher Scientific, Waltham, MA, USA, #AS153) containing desired concentrations of MeHg (methylmercury(II) chloride, Sigma-Aldrich, St. Louis, MO, USA, #442534, prepared in DMSO at 50 mM), HgCl_2_ (mercury(II) chloride, Sigma-Aldrich #215465), As (sodium (meta)arsenite, Sigma-Aldrich #S7400), and Pb (lead(II) acetate trihydrate, Sigma-Aldrich #467863), with four replicate vials of *n* = 50 larvae in each vial. Dose ranges for each metal were established empirically, based on the minimal and maximal effect seen across all the endpoints measured from prior *Drosophila* experiments [20,21,22,44]. Once per day, vials of developing flies were scored for pupa formation, pharate adult formation, and eclosion of adult fly for three of the four vials until these values in all vials were stagnant for 3 consecutive days, representing the endpoint. Thus, each experiment could end on a different day. From the fourth vial, yellow- to orange-eyed pupae were collected and pooled (*n* = 10) for metal analysis. For samples containing only MeHg and/or HgCl_2_, total Hg was determined by a DMA-80 Direct Mercury Analyzer (Milestone, Shelton, CT, USA). For As and Pb treatments, including mixtures containing MeHg, pupae collected were dissolved in high purity nitric acid at 90 °C for 2 h, and all metal levels were analyzed by ICP-Mass spectrometry on a Perkin-Elmer 2000C instrument. 

Our developmental time course collected two general types of data: (1) the number of individuals (expressed as a percent of total) that successfully reached the indicated endpoint, and (2) the time (in increments of days) taken to reach that endpoint. Endpoints scored consisted of: pupariation (formation of the white pre-pupae), metamorphosis (formation of the dark pupae or pharate adult), and eclosion (complete emergence of the adult from the pupal case). Graphs showing pupariation progression over time were created from the number of pupae formed at each timepoint expressed as a percentage of total (*n* = 50/vial) and averaged over the three replicate vials. For pupa metamorphosis and adult eclosion time courses, values were expressed as a percentage of the number of animals present at the initiation of the stage (e.g., number of white pupae at the initiation of metamorphosis and the number of dark pupae for the initiation of eclosion). These values were subsequently reported as the mean and standard deviation (SD) graphed over time using GraphPad Prism software. Pupariation time (PT), metamorphosis time (MT), and eclosion time (ET) were calculated as the time between the initiation and completion of the respective stage (PT initiates with larval loading on food; MT initiates with formation of white pupae, ET initiates with formation of dark pupae) and scored in each vial in increments of days. For MT, this was achieved by averaging the total time between loading larvae and formation of dark pupae (time to metamorphosis completion). Next, the average pupariation times (PT) for these same vials were subtracted from their average time to metamorphosis completion to give the MT represented in graphs. Eclosion time (ET) was calculated in a similar fashion by first averaging the total time from larval loading to eclosion for three replicate vials, then subtracting the average time to metamorphosis completion for these same vials to give the ET reported. A final scoring of the distribution of individuals across all endpoints (expressed as a percentage of total) with respect to metal concentration was performed. Individuals that had not reached the pupariation endpoint were designated to be stalled in the larval stage. Mean and SD used for statistics and graphs were gleaned from averaging the percentage in each stage from each of the three vials.

### 4.3. Statistics

Statistics were performed using JMP Pro 15 software, Cary, NC, USA. On all endpoints expressed as percentages, we performed logit transformation to improve the application of linear regression models. In the single metal studies, two-way analysis of variance analysis (ANOVA) was used to determine whether each developmental transition was delayed and whether there were differences in the endpoints between flies reared with and without metal exposure. Post hoc, we applied Tukey HSD to assign significance to metal-induced delays and lethality with significance for adjusted *p*-values less than 0.05. 

When comparing the four single-metal experiments, analysis of covariance analysis (ANCOVA) with Tukey post hoc comparisons was used to determine whether there was an interaction between the metal being analyzed and the dose or body burden of that metal on the outcomes of pupariation time and pharate adult eclosion (logit transformed). No interaction would indicate that the metals have the same effect over the dose or body burden. A significant interaction of *p* < 0.05 would suggest that the metals differ in effect by dose. 

ANCOVA with Tukey post hoc comparisons was also used to determine whether the metal mixture experiments showed interactions between metals. If the interaction term showed significance with a *p*-value less than 0.05, we would designate antagonism or synergy as determined by qualitative assessment. Additional analyses were performed using methods of Bliss and Loewe as previously described [45,46]. Otherwise, we would designate these metals as having no effect on each other or an additive dose effect as determined by analyzing effects by ANOVA with Tukey post hoc. If the effect of any of the concentrations tested was impacted by addition of the other metal, the effect was designated additive. 

We determined differences in eclosion between mixture conditions of MeHg and HgCl_2_ via two-way ANOVA with Tukey post hoc. If *p*-values less than 0.05 were found between values at the same HgCl_2_ concentration, the difference was considered significant.

## 5. Conclusions

The experimental model detailed here was able to identify developmental toxicity phenotypes of four metals and investigate the interactions of these metals when mixed together. We highlighted the exceptional potency of MeHg and its ability to uniquely inhibit eclosion failure. It was also observed that the potency was caused by toxicokinetics of absorption, and potency was equivalent to HgCl_2_ and As when adjusted for internal dose (body burden) with respect to pupariation delay. We next identified HgCl_2_ to be a potent antagonist to MeHg toxicity. To our knowledge, this is the first such report of testing for mixture interactions in such a controlled experimental model. Our methods provide a comprehensive way to identify relevant concentrations of the toxicants of interest and to design and analyze a mixture experiment with easy to score indicators of developmental toxicity. We anticipate these findings, and this methodology, will have important implications for future studies of unique mechanistic targets of MeHg and other toxicants to be evaluated in a mixture paradigm.
